# Optimization and evaluation of a non-invasive tool for peste des petits ruminants surveillance and control

**DOI:** 10.1038/s41598-019-41232-y

**Published:** 2019-03-18

**Authors:** Arnaud Bataille, Olivier Kwiatek, Salima Belfkhi, Lucile Mounier, Satya Parida, Mana Mahapatra, Alexandre Caron, Chobi Clement Chubwa, Julius Keyyu, Richard Kock, Bryony A. Jones, Geneviève Libeau

**Affiliations:** 1 CIRAD, UMR ASTRE, F-34398 Montpellier, France; 2 ASTRE, Univ Montpellier, CIRAD, INRA, Montpellier, France; 3 The Pirbright Institute, Ash Road, Pirbright, Woking, Surrey, UK; 4 CIRAD, UMR ASTRE, RP-PCP, Maputo, Mozambique; 5 Faculdade de Veterinaria, Universidade Eduardo Mondlane, Maputo, Mozambique; 6 Ngorongoro District Council, Arusha, Tanzania; 7 Tanzania Wildlife Research Institute, Arusha, Tanzania; 8 Royal Veterinary College, University of London, Hatfield, UK

## Abstract

Peste des petits ruminants (PPR) is a highly contagious and devastating viral disease affecting mainly sheep and goats, but also a large number of wild species within the order Artiodactyla. A better understanding of PPR transmission dynamics in multi-host systems is necessary to efficiently control the disease, in particular where wildlife and livestock co-occur. Notably, the role of wildlife in PPR epidemiology is still not clearly understood. Non-invasive strategies to detect PPR infection without the need for animal handling could greatly facilitate research on PPR epidemiology and management of the disease in atypical hosts and in complex field situations. Here, we describe optimized methods for the direct detection of PPR virus genetic material and antigen in fecal samples. We use these methods to determine the detection window of PPR in fecal samples, and compare the sensitivity of these methods to standard invasive sampling and PPR diagnostic methods using field samples collected at a wildlife-livestock interface in Africa. Our results show that quantitative reverse transcription PCR (RT-QPCR) amplification of PPRV from fecal swabs has good sensitivity in comparison to ocular swabs. Animals infected by PPRV could be identified relatively early on and during the whole course of infection based on fecal samples using RT-QPCR. Partial gene sequences could also be retrieved in some cases, from both fecal and ocular samples, providing important information about virus origin and relatedness to other PPRV strains. Non-invasive strategies for PPRV surveillance could provide important data to fill major gaps in our knowledge of the multi-host PPR epidemiology.

## Introduction

Peste des petits ruminants (PPR) is a highly contagious and devastating disease caused by a virus of the genus *Morbillivirus* in the family *Paramyxoviridae*^1^. PPR virus (PPRV) affects mainly sheep and goats, but also a large number of wild species within the order Artiodactyla^1,2^. PPR occurrence in livestock must be notified to the World Organization for Animal Health (OIE) and the disease is now the target of a global eradication campaign^3^. PPR is endemic in large parts of Africa, the Middle East and Asia, and is still spreading globally, with emergence notably reported in Georgia^4^, Mongolia^5^, and most recently within the European Union in Bulgaria^6^.

Better understanding of PPR transmission dynamics is necessary to efficiently control the disease. Notably, the role of wildlife in PPR epidemiology is still not clearly understood^7,8^. Importantly, the potential for spill-over PPR infection between wildlife and livestock and the direction of these spill-overs are still poorly studied. Also, the capacity of PPR virus to be transmitted and maintained within wildlife populations without a domestic host source has still not yet been demonstrated. The role of wildlife is particularly important to explore in Africa and Central-South Asia including the Himalayas where multi-host systems composed of a large diversity and population of wild ungulate species and domestic populations co-occur. A range of wildlife-livestock interfaces provides ample opportunities for virus sharing between wild and domestic hosts. So far, the knowledge about the role of wildlife in PPR epidemiology, recently reviewed^8,9^, is limited to outbreaks from *ex-situ* populations in zoos and fenced enclosures, and to rare recent *in situ* epidemics affecting mountain goats^10–13^ and, on one occasion, free-ranging antelope and wild caprines in Mongolia^14^. Transmission of PPR between domestic and non-domestic species could hinder control of the disease in some areas. Importantly, recent mass mortality of saïga antelopes in Mongolia^15^ due to PPR infection showed that this disease also represents a serious threat to some critically endangered wildlife populations^14^.

Despite the need for more data, wildlife disease surveys are rare because animal capture is very costly and requires complicated logistics in remote areas. Permits for protected animals are also difficult to obtain for invasive sampling and sample transportation across borders. Even for domestic animals, it may be hard to obtain samples in some regions if farmers are reluctant to have their animals handled and tested for PPR infection. Non-invasive strategies to detect PPR infection without the need for animal capture and handling could greatly facilitate research on PPR epidemiology in atypical hosts (e.g. wildlife) and in complex field situations.

Non-invasive samples, notably fecal samples, have been used to study wildlife ecology and health for more than two decades^16^. Fecal samples are now also being used to explore virus diversity in wildlife^17,18^. Several studies have shown that PPR antigen and RNA can be detected in feces of infected goats for weeks after infection^19–21^. However no study so far has looked into optimizing diagnostic methods for PPR detection in fecal material and compared the sensitivity of such methods with standard diagnosis from invasive sampling. Indeed, robust commercial serological and virological diagnostic kits are available to detect PPR infection, but they were mainly developed for domestic small ruminants (goat and sheep) and for high quality, invasive samples. These methods need to be adapted for PPR virus detection from fecal samples.

Here, we describe optimized methods for the detection of PPR virus genetic material and antigen in fecal samples. We use these methods to determine the detection window of PPR virus in fecal samples, and compare the sensitivity of these methods to standard invasive sampling and PPR diagnostic methods using field samples collected in Africa at the wildlife-livestock interface.

## Results

### Optimization of PPR antigen detection in fecal samples

Preliminary tests performed with fecal samples spiked with serially diluted PPR vaccine showed that fecal samples contained inhibitors that lowered the sensitivity of reverse transcription Polymerase Chain Reaction (RT-PCR) and Enzyme-linked immunosorbent (ELISA) assays used for the diagnosis of PPR (see Supplementary Information). Viral RNA extraction with magnetic beads increased the sensitivity of downstream quantitative RT-PCR assays (RT-QPCR), compared to a column-based RNA extraction approach. Our preliminary tests also showed that some RT-PCR kits appeared to be less sensitive to inhibitors from fecal samples than others. Increasing incubation time for antigen capture ELISA (AgELISA) assay on fecal samples also increased the sensitivity of the assays without compromising specificity. Optimized protocols were developed to detect PPRV in fecal samples using RT-QPCR, RT-PCR, and AgELISA based on these preliminary results (see Supplementary Information).

### Detection window of PPR viral antigen in rectal fecal material

**Figure 1:**
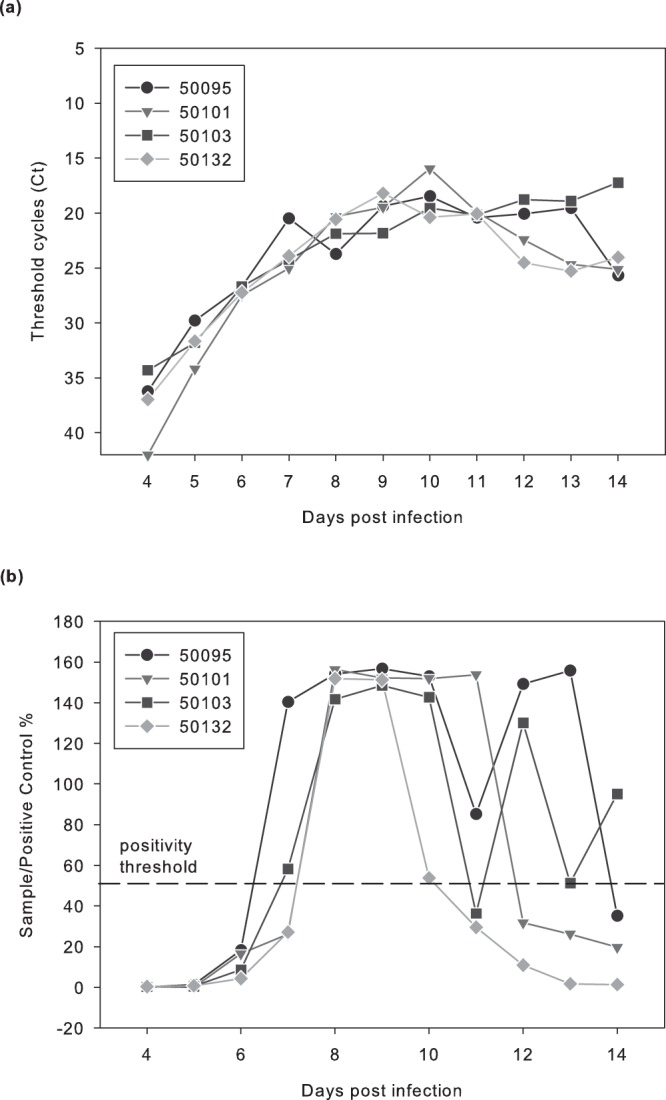
Kinetics of viral shedding in fecal material of PPRV infected goats. Viral shedding was monitored by **(a)** RT-QPCR (expressed in Ct, limit of detection = 40 Ct) and **(b)** antigen capture ELISA (expressed in Sample/Positive Control % following the manufacturers’ instructions, limit of detection = 20%). Samples were collected from four Saanen goats infected by an intranasal route with the highly virulent Peste des Petits Ruminants strain Morocco 2008^22^. Fecal material was tested for presence of PPRV genetic material from day 4 post infection (dpi) until the end of the experiment. ID numbers used in Figure label indicates animal ID numbers.

We evaluated the time window of PPR excretion in rectal fecal material using rectal swabs collected daily from day 0 post infection (dpi) to 14 dpi during a previously described experimental challenge on Saanen goats^22^. We focused on samples collected from four individuals infected by an intranasal route with a highly virulent PPR strain (Morocco 2008) as this route of infection simulates natural infection. PPRV was detected in rectal swabs from 5 dpi until the end of the experiment (14 dpi) using our optimized RT-QPCR method (Fig. 1). PPRV was also detected in fecal swabs by antigen capture ELISA, but the first positive result was obtained at 6 dpi, with strong optical density values obtained from 7 dpi. Antigen capture ELISA results became negative at 12 dpi for one individual, while rectal swabs taken at 12 to 14 dpi from the other 3 animals were still positive, although close to the threshold (Fig. 1).

### PPR viral antigen detection on field fecal samples

**Figure 2:**
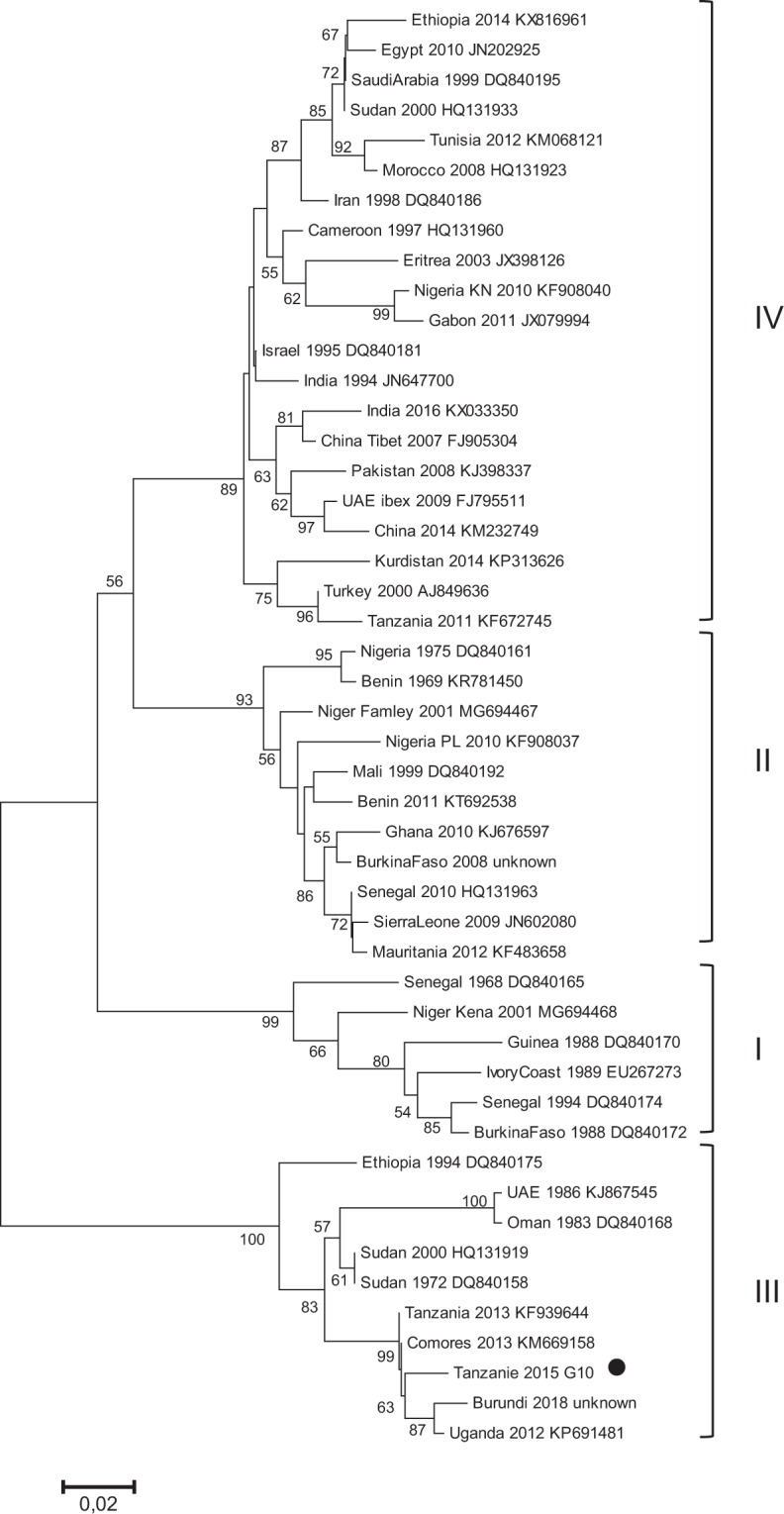
PPRV N gene phylogenetic analysis. Neighbour-joining tree showing the relationship between the partial N gene sequence obtained in this study (indicated by black dot) and sequences publically available in GenBank (indicated by the country and sample name, the year and the GenBank Accession Number). The numbers at the nodes are bootstrap values obtained from 1000 replicates. Only bootstrap values > 50 are shown.

As part of a larger study on the role of wildlife in PPR transmission, suspected cases of PPR were investigated in domestic sheep and goat flocks kept in close proximity to wild herbivores in the Ngorongoro district in the Serengeti ecosystem of northern Tanzania in 2015. Conjunctival swabs were collected from animals with clinical signs of PPR infection (see Table S1 in Supplementary Information), and tested in the field for presence of PPR antigen using the lateral flow device (LFD) Peste-Test Rapid Field Test for PPRV (BDSL Irvine, UK). Further conjunctival and nasal swabs were tested in the laboratory of the Pirbright Institute for the presence of PPRV genetic material using published RT-PCR^23^ and RT-QPCR^24^ methods targeting a portion of the PPR N gene. If present, fecal samples were collected by gloved hand from the rectum of the same animals, and were tested for presence of PPRV using RT-QPCR, RT-PCR and AgELISA methods optimized during this study. We analysed fecal samples collected from five goats and six sheep. Viral genetic material could be detected in fecal samples by RT-QPCR in all goats tested positive by RT-PCR and RT-QPCR on ocular swabs (Table 1). Only one out of six sheep (animal S14) was found positive by PCR and LFD on ocular swabs whereas all sheep fecal samples remained negative. Only two fecal samples that tested positive by RT-QPCR were found positive by RT-PCR and AgELISA. These two samples had relatively high Ct values (Ct: 23–27) by RT-QPCR (Table 1). The PCR products obtained after RT-PCR were sequenced and aligned to publicly available partial N gene sequences to identify the genetic lineage using phylogenetic analysis. Sequences retrieved from ocular swabs and fecal samples were identical (Genbank accession number MK201803), and were positioned within the lineage III of PPRV in the phylogenetic tree (Fig. 2).

**Table 1:** Summary of results obtained from multiple diagnostic methods for PPRV genetic material or antigen with ocular swabs or fecal material.

	Sample code	Ocular swabs			Feces		
		PCR	QPCR	LFD antigen test	PCR	QPCR	AgELISA
Goat	G4	+	no Ct	+	+	26.9	+(68%)
	G10	+	22.96	+	+	22.92	+(120%)
	G11	+	36.96	+	−	35.64	−(<20%)
	G16	n.d.	26.37	n.d.	−	26.9	−(<20%)
	G74^§^	−	no Ct	n.d.	n.d.	no Ct	−(<20%)
Sheep	S14	+	no Ct	+	n.d.	no Ct	−(<20%)
	S19	n.d.	no Ct	n.d.	n.d.	no Ct	−(<20%)
	S20	n.d.	no Ct	n.d.	n.d.	no Ct	−(<20%)
	S31	n.d.	no Ct	−	−	no Ct	−(<20%)
	S33	−	no Ct	n.d.	n.d.	no Ct	−(<20%)
	S37*	n.d.	no Ct	n.d.	n.d.	no Ct	−(<20%)
Total	11	6	11	5	5	11	11
Total positive		4	3	4	2	4	2

Results for reverse transcription Polymerase Chain Reaction (PCR) and lateral flow device (LFD) antigen test are indicated as positive (+) or negative (−) status. Results for quantitative reverse transcription Polymerase Chain Reaction (QPCR) are presented in Ct values, with lower Ct values indicating presence of higher amount of target PPR genetic material and “no Ct” indicating no detection of PPR genetic material. Results of the antigen capture ELISA (AgELISA) are presented as positive (+) or negative (−) status, with OD values converted to Sample/Positive Control % following the manufacturers’ instructions between brackets. Tissue from oral lesions (*) or post-mortem samples (^§^) were collected instead of ocular swabs in some occasions; n.d., analysis not done.

## Discussion and Conclusion

Non-invasive samples, especially fecal samples, could represent a very useful tool to detect PPR in wildlife and, to a lesser extent, domestic ruminants from remote and complex wildlife-livestock interfaces. Easier and cheaper sampling in complex field situations would provide the means to fill in major gaps in our knowledge of PPR epidemiology, increasing our chances of successful eradication of this disease^25,26^. Our results based on infection experiments and domestic animals at the wildlife-livestock interface suggest that fecal samples may offer a good alternative to standard ocular or nasal swabs, when such invasive sampling is difficult to obtain.

Results from infection experiments suggest that shedding of PPR virus in feces starts five days after infection. Ocular excretion of the virus starts three days earlier^22^, so fecal samples have some disadvantages in this regard. However, PPRV genetic material could be detected in rectal swabs by RT-QPCR until the end of the experiment, confirming results obtained in previous studies^19,20^ and suggesting that animals infected by the virus could be identified relatively early on and during the whole course of infection based on fecal samples.

We put in place optimized protocols to detect PPR genetic material and antigens in fecal samples despite the presence of inhibitors. These protocols may be implemented in any laboratory with standard equipment. Samples obtained from domestic animals at the wildlife-livestock interface in the Serengeti ecosystem in northern Tanzania gave us the opportunity to test our protocols in a field situation. RT-QPCR results from fecal samples were similar to results obtained from ocular swabs, confirming that high quantity of virus genetic material can be retrieved in the field from feces of infected animals. PPRV RNA could be detected in ocular swabs of one sheep whereas RT-QPCR on fecal samples from the same animal gave negative results. It is possible that sampling was performed on this animal when virus was shed in ocular excretions but not yet in fecal excretions. The fecal sample may also have been degraded during transport. These results may also suggest that the issue of PCR inhibition in fecal samples, although reduced with our optimized method, has not been completely resolved. In all cases, these potential issues have to be kept in mind when interpreting diagnostic results.

PPRV RNA and antigen could also be retrieved from fecal material using RT-PCR and antigen capture ELISA. However, sensitivity of these two methods is lower than RT-QPCR. Also, the window of detection for antigens appears to be shorter and more variable, probably due to production of antibodies by infected animals. Still, these methods may be a useful addition to the RT-QPCR method. RT-PCR is especially interesting, as our results show that partial gene sequence can be retrieved from fecal material after amplification, providing important information about virus origin and relatedness to other PPRV strains^27^. In this study, sequencing results obtained from both ocular swabs and fecal sample confirm the circulation in 2015 of the lineage III of PPRV in the Northern Tanzania. This lineage has been present in the region for several years^7,28^. Most likely, full genome of PPRV could also be retrieved from fecal samples of infected animals, although concentration and quality of viral RNA would need to be high^29^.

In our field study, fecal material was directly collected from the rectum, possibly increasing our chances to detect PPRV material from rectal epithelium. Capacity of PPRV detection from excreted fecal material is likely to be more variable, but this study shows that chances are high to obtain virus sample from feces of infected animals without the need to catch them. However, fresh fecal samples would need to be collected to ensure a good quality of viral RNA. In addition, PPRV is quickly inactivated in the environment^30^, so fecal samples needs to be retrieved within 1–2 hours of excretion if one hopes to detect PPRV genetic material from this source. Thus fecal sampling for PPRV detection still involves particular logistics to follow closely animals and ensure collection of fresh samples and to have the necessary means to store the samples under optimal cold storage conditions until shipment to a laboratory. This being said, carefully designed protocols on wildlife species implemented in collaboration with staff trained in wildlife ecology and/or management could easily ensure fresh collection of fecal samples after visual observation of the deposition, together with characteristics of the sampled individuals such as gender, age, body condition and reproductive status, which could prove important to relate PPRV infection to host traits. The choice of individuals and herds to sample should also be based on observation of clinical signs or on epidemiological data suggesting circulation of PPR in the area (for example, outbreak in domestic animals).

This study offers the first research data on how fecal samples could be exploited for non-invasive PPR detection. Past attempts by our laboratory to detect PPR antibodies in fecal samples have failed so far (data not shown), suggesting that non-invasive strategies for PPR serological surveys may be harder to develop. On the other hand, some trials suggests that penside tests can detect PPR antigen in feces of domestic small ruminants^31^, but also of wildlife such as saiga antelopes (R. Kock, personal communication). Implementing such tests in a routine manner would permit fast non-invasive diagnosis of PPR infection in the field, and therefore quick response of veterinary and wildlife services. PPR has a major socio-economic impact across many regions of the globe^32^, and represents a threat to some endangered wildlife^2^. We will need all the tools available to better understand and control the disease. Non-invasive strategies to sample PPR represent an important addition to this toolbox.

## Material and Methods

### Fecal samples from suspected cases of PPR in the field

Reports of suspected PPR cases in domestic mixed sheep and goat flocks in Ngorongoro District of northern Tanzania were investigated. Animals with clinical signs of pyrexia, lacrimation, nasal discharge and/or mouth lesions were selected for diagnostic testing. Sampling was designed in accordance to guidelines and regulations of the Royal Veterinary College Ethics Committee and was performed following approval by the same committee (Approval number 2015 1326). Conjunctival swabs were collected from animals with pyrexia and tested by PPR rapid diagnostic test. Conjunctival and nasal swabs were collected for conventional^23^ and quantitative PCR^24^ assays at the Pirbright Institute. Fecal samples were collected by rectal examination from animals which had feces present in the rectum. All samples were put into a cool box with ice packs for transportation to the field base where they were stored in a freezer at −20C, until further transportation by cool box to the TAWIRI laboratory in Arusha where they were stored at −80C. Samples were shipped to Pirbright and CIRAD in dry ice.

### Optimization of PPRV diagnostic from fecal samples

Details of the methods used to detect and reduce the effect of inhibitors and increase sensitivity of RT-PCR, RT-QPCR, and ELISA assay performed on rectal swabs and fecal samples to detect the presence of PPR virus are provided as Supplementary Information. The optimized protocol is described below.

### Fecal sample preparation

For RT-PCR, fecal samples were ground in 3 ml of Minimum Essential Media (MEM, 10% W/V) with 0.2 μm glass beads, and then centrifuged 3 min at 1000 g to collect supernatant. RNA was extracted from 150 μl of supernatant using the ID gene MAG universal extraction kit (IDvet genetics, Grabels, France) and a KingFisher automated extractor (ThermoFisher, IDvet genetics, France), following the manufacturers’ instructions. For the antigen capture ELISA, fecal sample were ground in 3 ml (10% W/V) of the buffer 13 of the IDscreen PPR antigen capture ELISA kit (IDvet, Grabels, France) with 0.2 μm glass beads and then centrifuged 3 min at 1000 g to collect supernatant.

### Detection of PPR genetic material in fecal samples

A one-step RT-QPCR method was used to amplify the partial end of the N protein gene^33^, with the qscript XLT kit one-step RT-qPCR ToughMix (Quantabio, VWR, Fontenay-sous-Bois, France). The amplification cycle consisted of a reverse transcription step of 45 °C for 10 min, an initial denaturation at 95 °C for 10 min, followed by 40 cycles of 95 °C for 15 s and 60 °C for 45 s. Serially diluted standard controls were included in the RT-QPCR runs to validate the test and obtain an estimation of the number of copy of PPR N protein in the samples. The RT-QPCR runs were performed on a LightCycler instrument (Roche, IDvet genetics, Montpellier, France). A RT-PCR was performed on samples found positive by RT-QPCR analysis. The qScript XLT One-Step RT-PCR Kit (Quantabio, VWR, Fontenay-sous-Bois, France) was used to amplify a 351 base pair (bp) segment of the PPRV N gene with the NP3/NP4 (Forward NP3: 5′-GTC-TCG-GAA-ATC-GCC-TCA-CAG-ACT-3′ and Reverse NP4: 5′-CCT-CCT-CCT-GGT-CCT-CCA-GAA-TCT-3′) diagnostic primers modified from^23^. The amplification cycle consisted of a reverse transcription step of 48 °C for 20 min, an initial denaturation at 94 °C for 3 min, followed by 40 cycles of 94 °C for 15 s and 60 °C for 30 sec and a final extension step at 72 °C for 1 min. PCR products were resolved on a 1.5% agarose gel to reveal the expected band size. Positive and negative controls were included in the RT-PCR runs to validate the test.

### Enzyme-linked immunosorbent assay (ELISA) on fecal samples

Fecal samples were tested for presence of viral particles using the IDscreen PPR antigen capture ELISA (IDvet, Grabels, France). The assays were performed and analyzed following the manufacturer’s instructions except for the incubation step that was done overnight at room temperature to maximize the sensitivity of the test (see Supplementary Methods). Optical density (OD) values at 450 nm were recorded with a Sunrise ELISA reader (Tecan, Lyon, France). OD values were converted to S/P % following the manufacturers’ instructions. According to the cut-off value of the test, test samples with S/P values ≥ 20% were considered positive. Presence of PPR antibodies in fecal samples was tested using the IDscreen PPR competition ELISA (IDvet, Grabels, France). The assays were performed and analyzed following the manufacturer’s instructions except for the incubation step that was done overnight at room temperature to maximize the sensitivity of the test (see Supplementary Methods). OD values were converted to percent competition (PC). According to the cut-off value of the test, test samples with PC values ≤ 50% were considered positive.

## Supplementary information

Supplementary information

## Data Availability

All data generated or analysed during this study are included in this published article (and its Supplementary Information files).
