# Supplementary material for: Optimization and evaluation of a non-invasive tool for peste des petits ruminants surveillance and control
Source: Sci Rep. 2019 Mar 18;9:4742. doi: 10.1038/s41598-019-41232-y (PMC6426962; doi:10.1038/s41598-019-41232-y)
Supplement: Supplementary file 1 — Supplementary information [file 41598_2019_41232_MOESM1_ESM.docx]

**Optimization and evaluation of a non-invasive tool for peste des petits ruminants surveillance and control**

Arnaud Bataille^1,2*^, Olivier Kwiatek^1,2^, Salima Belfkhi^1,2^, Lucile Mounier^2^, Satya Parida^3^, Mana Mahapatra^3^, Alexandre Caron^2,4,5^, Chobi Clement Chubwa^6^, Julius Keyyu^7^, Richard Kock^8^, Bryony A Jones^8^, Geneviève Libeau^1,2^

^1^ CIRAD, UMR ASTRE, F-34398 Montpellier, France

^2^ ASTRE, Univ Montpellier, CIRAD, INRA, Montpellier, France.

^3^  The Pirbright Institute, Ash Road, Pirbright, Woking, Surrey, UK.

^4^ CIRAD, UMR ASTRE, RP-PCP, Maputo, Mozambique

^5^ Faculdade de Veterinaria, Universidade Eduardo Mondlane, Maputo, Mozambique

^6^ Ngorongoro District Council, Arusha, Tanzania

^7^ Tanzania Wildlife Research Institute, Arusha, Tanzania

^8^ Royal Veterinary College, University of London, Hatfield, UK.

*Corresponding author: arnaud.bataille@cirad.fr

**Supplementary material**

**Detection of PCR inhibitors in fecal samples**

Fecal samples were collected from PPR-negative goats kept in the animal facility of CIRAD (Montpellier, France). Samples were ground in 3ml of Minimum Essential Media (MEM, 10% W/V) with 0.2μm glass beads, and then centrifuged 3 min at 1000g to collect supernatant. RNA was extracted from 150μl of supernatant using the NucleoSpin RNA virus extraction kit (Macherey-Nagel, Hoerdt, France) following the manufacturer’s instructions. RNA from the PPR vaccine strain Nigeria 75/1 was extracted using the same protocol. PPR RNA was serially diluted to 1/5^th^, 1/25^th^, 1/125^th^ and 1/625^th^ using on one hand molecular grade water, and on the other hand RNA extracted from the fecal samples.

A reverse transcription Polymerase Chain Reaction (RT-PCR) was performed with 2µl of both sets of spiked samples using the Qiagen one step RT-PCR (Qiagen, Germany) to amplify a 351 base pair (bp) segment of the PPRV N gene with the NP3/NP4 (Forward NP3: 5’-GTC-TCG-GAA-ATC-GCC-TCA-CAG-ACT-3’ and Reverse NP4: 5’-CCT-CCT-CCT-GGT-CCT-CCA-GAA-TCT-3’) diagnostic primers^1^. PCR was set up under the following conditions: 50°C for 30 min; 95°C for 15 min and 40 cycles of amplification (10sec at 95°C, 30sec at 60°C and 30sec at 72°C) and a final extension step at 72°C for 5min. PCR products were resolved on a 1.5% agarose gel to reveal the expected band size. Results showed that a clear band of the expected size could be obtained at the dilution 1/125^th^ in water but not in fecal extraction eluate (Figure S1), suggesting presence of inhibitors in eluate from fecal samples.

**Comparison of RNA extraction methods**

Fecal samples were spiked with PPR vaccine strain Nigeria 75/1 diluted in MEM to 10^-3^, 10^-4^, 10^-5^, and 10^-6^. RNA was extracted from the spiked samples, serially diluted virus isolates and a selection of PPR-positive organ, swab, and blood samples using (i) a column-based method: the NucleoSpin® 96 Virus core kit (Macherey-Nagel, Hoerdt, France) and a BIOMEK automated extractor (Beckman Coulter, Villepinte, France); (ii) a magnetic beads method: the ID gene MAG universal extraction kit (IDvet genetics, Montpellier, France) and a KingFisher automated extractor (ThermoFisher, IDvet genetics, Montpellier, France), following the manufacturers’ instructions.

A one-step method reverse transcription followed by quantitative polymerase chain reaction (RT-QPCR) was used to amplify the partial end of the N protein gene ^2^, with the qscript XLT kit one-step RT-qPCR ToughMix (Quantabio, VWR, Fontenay-sous-Bois, France). The amplification cycle consisted of a reverse transcription step of 45°C for 10 min, an initial denaturation at 95°C for 10 min, followed by 40 cycles of 95°C for 15 s and 60°C for 45 s. Serially diluted standard controls were included in the RT-QPCR runs to validate the test and obtain an estimation of the number of copy of PPR N protein in the samples. The RT-QPCR runs were performed on a LightCycler instrument (Roche, IDvet genetics, Montpellier, France). The results showed that sensitivity of detection was increased when samples were extracted using magnetic beads (Table S2). It was especially evident with organ and blood samples, as no PPRV RNA could be detected using a column-based extraction method, whereas relatively high RNA copy numbers were observed after magnetic beads extraction.

**Optimization of antigen capture ELISA on fecal samples**

We aimed at optimizing the detection of viral particles in fecal samples using the IDscreen PPR antigen capture ELISA (enzyme-linked immunosorbent assay) (IDvet, Grabels, France). Fecal samples spiked with PPR vaccine strain Nigeria 75/1 and pure PPR vaccine strain Nigeria 75/1 were serially diluted in buffer 13 of the kit or in MEM from 10^-1^ to 10^-6^. Antigen capture ELISA assay was performed following the manufacturer’s instructions except for the incubation step. Three conditions were tested for the incubation step: (i) incubation during 45min at 37°C; (ii) incubation over-night at room temperature (21±5°C); (iii) incubation over-night at 4°C. A Sunrise ELISA reader was used for reading at 450 nm (Tecan, Lyon, France). Optical density values were converted to S/P % following the manufacturers’ instructions. According to the cut-off value of the test, test samples with S/P values ≥ 20% were considered positive. Results show that use of buffer 13 for dilution and incubation over-night at room temperature increased the sensitivity of the method (Figure S2).

The antigen capture ELISA test with the best incubation condition was further validated on fecal samples of PPR-negative wild Artiodactyla species from Montpellier zoo. This step was necessary to ensure that there was no risk of false positive when using the new optimized incubation conditions. First, serum samples collected from 21 wild Artiodactyla individuals (see table S3) by zoo staff were tested for the presence of PPRV antibodies using the IDscreen PPR competition ELISA (IDvet, Grabels, France). The assays were performed and analyzed following the manufacturer’s instructions except for the incubation step that was done overnight at room temperature to maximize the sensitivity of the test (see supplementary methods). Optical density (OD) values at 450 nm were recorded with a Sunrise ELISA reader (Tecan, Lyon, France). OD values were converted to percent competition (PC). According to the cut-off value of the test, test samples with PC values ≤ 50% were considered positive. Results confirmed that all the animals had never been in contact with PPR. Secondly, fecal samples, collected from the same individuals by the zoo staff, were tested for presence of PPR virus using the antigen capture ELISA test, following the protocol optimized in our laboratory. All results were negative, confirming that the specificity of the kit is maintained when used with wildlife fecal samples and modified incubation protocol (Figure S3).

**Comparison of RT-PCR methods**

RT-PCR kits may differ in their sensitivity to PCR inhibitors present in fecal samples. On fecal samples collected in the field and found PPR-positive by RT-QPCR (see main text), we applied two different RT-PCR kits. Primers used were NP3/NP4 primers targetting a 351 base pair (bp) fragment belonging to the PPRV N gene. Qiagen one step RT-PCR (Qiagen, Germany) was first tested. PCR was set up under the following conditions: 50°C for 30 min; 95°C for 15 min and 40 cycles of amplification (10sec at 95°C, 30sec at 60°C and 30sec at 72°C) and a final extension step at 72°C for 5min. For the second kit tested, qScript_XLT_One-Step_RT-PCR Kit (Quantabio, VWR, Fontenay-sous-Bois, France), the amplification cycle consisted of a reverse transcription step of 48°C for 20 min, an initial denaturation at 94°C for 3 min, followed by 40 cycles of 94°C for 15 s and 60°C for 30 sec and a final extension step at 72°C for 1min. PCR products were resolved on a 1.5% agarose gel to reveal the expected band size. Positive and negative controls were included in the RT-PCR runs to validate the tests. Results show that an amplicon of the expected size could be obtained for one goat (G4), found positive by RT-QPCR (see main text) using the QuantaBio kit, but not the Qiagen kit (Figure S4). It suggests that the QuantaBio kit performs better for RT-PCR targeting the N gene of PPRV in fecal samples.

**Tables**

**Table S1.** Summary of clinical signs for animals from which field feces samples collected from goats and sheep in Ngorongoro District of northern Tanzania.

|  | Sample code | Age | Flock no. | Pyrexia (>40.0°C) | Lacrimation | Nasal discharge | Oral lesions | Respiratory signs | Diarrhoea |
| --- | --- | --- | --- | --- | --- | --- | --- | --- | --- |
| Goat | G4 | 1 yr | 1 | n.d. | No | Yes | Yes | No | No |
|  | G10 | 2 yr | 3 | Yes | Yes | Yes | No | No | Yes |
|  | G11 | 6 mth | 3 | Yes | Yes | Yes | Yes | No | No |
|  | G16 | 4 mth | 6 | No | No | Yes | No | No | Yes |
|  | G74 | 3 yr | 20 | No | No | No | No | Yes | No |
| Sheep | S14 | 2 yr | 5 | No | No | Yes | Yes | No | No |
|  | S19 | adult | 9 | No | No | Yes | No | No | No |
|  | S20 | 4 yr | 9 | No | No | Yes | Yes | No | Yes |
|  | S31 | 7 mth | 16 | Yes | No | Yes | No | No | Yes |
|  | S33 | 7 mth | 17 | Yes | No | Yes | Yes | No | No |
|  | S37 | 4 yrs | 18 | No | Yes | Yes | Yes | No | Yes |

n.d. – temperature not recorded

**Table S2.** Comparative results for a RT-QPCR (expressed in Ct, limit of detection = 40 Ct) targeting the PPRV N gene in RNA extracts obtained using either a column-based method or a magnetic beads method.

| **Sample** | **Magnetic beads extraction** | **Column-based extraction** |
| --- | --- | --- |
| Spiked feces (10^-3^) | 31.44 | 28.15 |
| Spiked feces (10^-4^) | 34.06 | 30.46 |
| Spiked feces (10^-5^) | no Ct | no Ct |
| Spiked feces (10^-6^) | no Ct | no Ct |
| Organ (Ghana_2004_1) | no Ct | 17.75 |
| Organ (Ghana_2004_2) | no Ct | 18.73 |
| Swab (Burkina Faso 2014) | 33.05 | 29.67 |
| Blood (Inf Exp 1) | no Ct | 26.98 |
| Blood (Inf Exp 2) | no Ct | 26.54 |

Samples tested were fecal samples of PPR-negative animals spiked with a PPRV vaccine strain (serially diluted), two organs and one swab from the CIRAD sample collection, and blood from animals infected by PPRV during an infection experiment (Inf Exp)^3^. All samples have been tested with both types of RNA extraction methods.

**Table S3.** List of animals from Lunaret zoo (Montpellier, France) sampled for serum and fecal material

| **ID code** | **Species name** | **Date of sampling** |
| --- | --- | --- |
| M05002 | Addax (*Addax nasomaculatus*) | 02/02/2017 |
| M05003 | Addax (*Addax nasomaculatus*) | 02/02/2017 |
| M05022 | Addax (*Addax nasomaculatus*) | 16/02/2017 |
| M08090 | Addax (*Addax nasomaculatus*) | 02/02/2017 |
| M14109 | Addax (*Addax nasomaculatus*) | 09/02/2017 |
| M15038 | Addax (*Addax nasomaculatus*) | 09/02/2017 |
| M15058 | Addax (*Addax nasomaculatus*) | 02/02/2017 |
| M16073 | Addax (*Addax nasomaculatus*) | 02/02/2017 |
| M04043 | Barbary sheep (*Ammotragus lervia*) | 02/02/2017 |
| M04042 | Barbary sheep (*Ammotragus lervia*) | 16/02/2017 |
| M05015 | Barbary sheep (*Ammotragus lervia*) | 09/02/2017 |
| M02005 | Barbary sheep (*Ammotragus lervia*) | 07/02/2017 |
| M10096 | Dama gazelle (*Nanger dama mhorr*) | 29/12/2016 |
| M10095 | Dama gazelle (*Nanger dama mhorr*) | 21/02/2017 |
| M06010 | Bharal (*Pseudois nayaur*) | 09/02/2017 |
| M06163 | Bharal (*Pseudois nayaur*) | 09/02/2017 |
| M15048 | Bharal (*Pseudois nayaur*) | 09/02/2017 |
| M15045 | Bharal (*Pseudois nayaur*) | 22/02/2017 |
| M13004 | Bharal (*Pseudois nayaur*) | 22/02/2017 |
| M14057 | Bharal (*Pseudois nayaur*) | 09/02/2017 |
| M15042 | Bharal (*Pseudois nayaur*) | 09/02/2017 |
| M15046 | Bharal (*Pseudois nayaur*) | 24/01/2017 |
| M13060 | Bharal (*Pseudois nayaur*) | 19/01/2017 |
| M07001 | Bongo (*Tragelaphus eurycerus*) | 25/01/2017 |
| M10097 | Bongo (*Tragelaphus eurycerus*) | 11/01/2017 |
| M15050 | Bongo (*Tragelaphus eurycerus*) | 24/03/2017 |
| M09101 | Beisa Oryx (*Oryx beisa*) | 15/03/2017 |
| M09102 | Beisa Oryx (*Oryx beisa*) | 14/03/2017 |
| M10098 | Beisa Oryx (*Oryx beisa*) | 14/03/2017 |
| M16071 | Nyala (*Tragelaphus angasii*) | 24/03/2017 |

**Figures**

**a)**

**
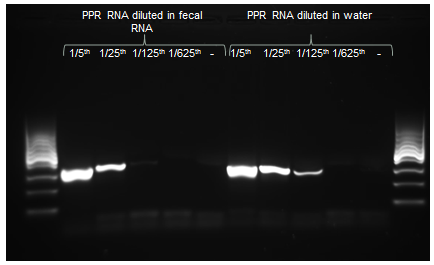
**

100bp

200bp

300bp

400bp

**b)**

**
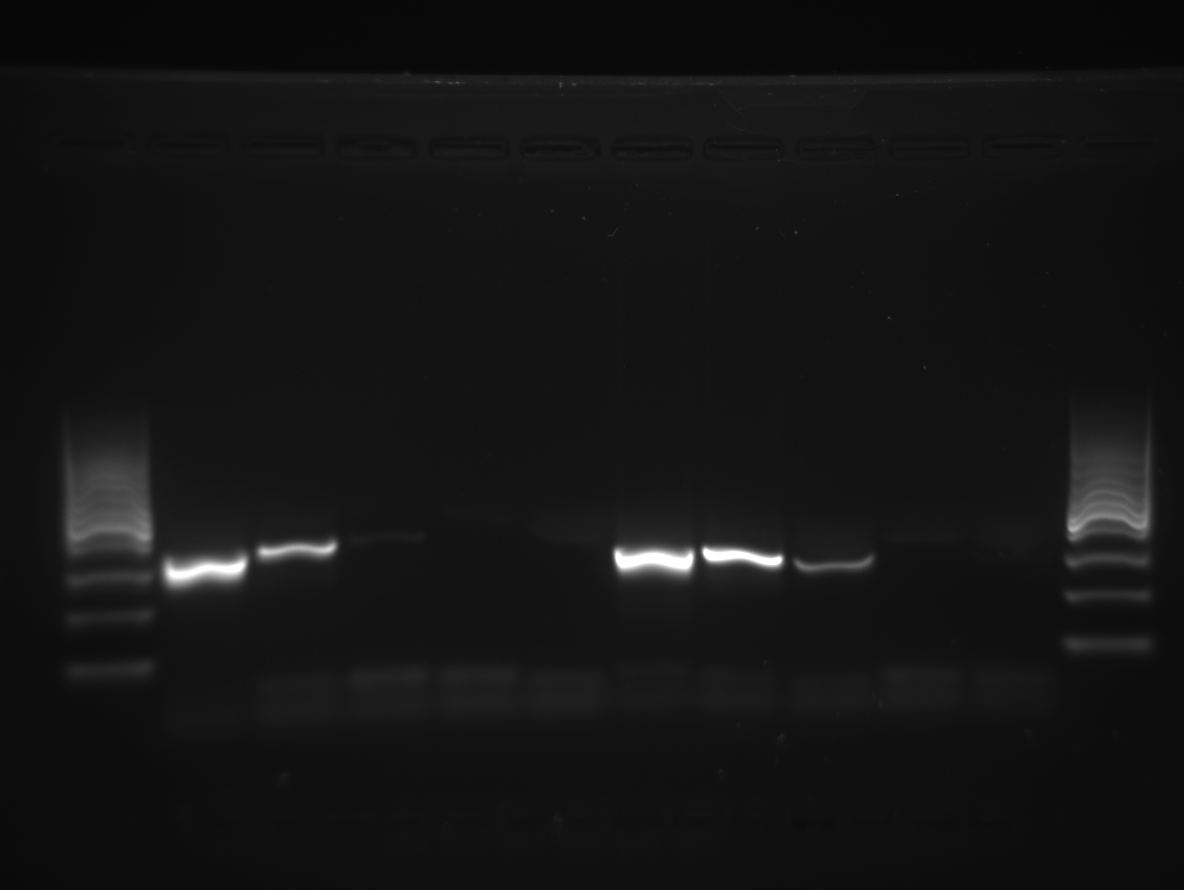
**

**Figure S1.** Comparative results for a RT-PCR amplifying a 351 base pair (bp) segment of the PPRV N gene when PPR RNA is serially diluted in water or in the eluate of an RNA extraction from PPR-negative fecal samples (a: annotated picture; b: original gel picture).

**
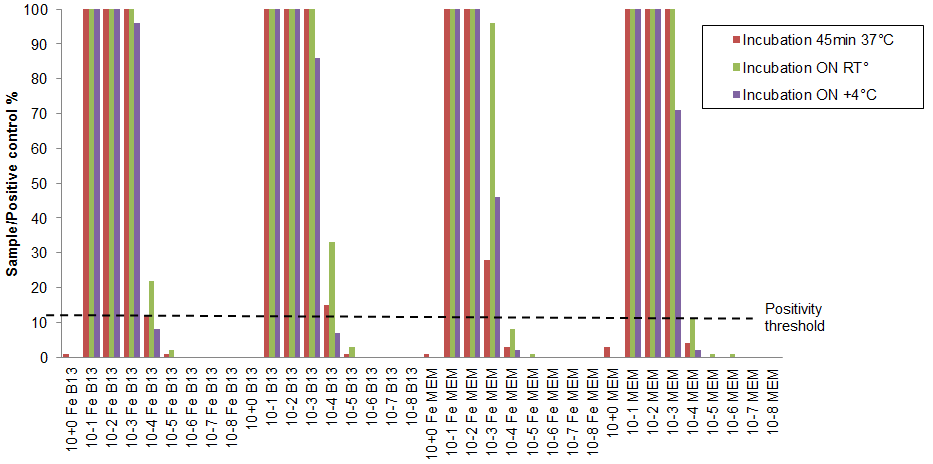
**

**Figure S2.** Comparative results for PPRV antigen capture ELISA (AgELISA, IDvet, France) using three different incubation protocols. Samples tested were fecal samples (Fe) spiked with serial dilutions of PPRV vaccine strain (Nigeria 75/1) or pure serially diluted PPRV vaccine strain. Serial dilutions were performed in either (i) Buffer 13 of AgELISA kit (B13) or (ii) in Minimum Essential Media (MEM). Results are expressed in Sample/Positive Control % following the manufacturers’ instructions (limit of detection = 20%). ON, over-night; RT, room temperature (21 ± 5°C).

**
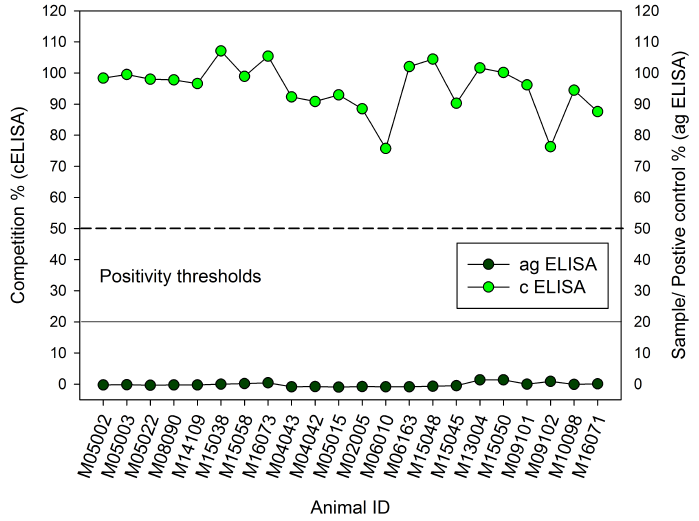
**

**Figure S3.** Detection of PPRV antigen and antibodies by antigen capture ELISA (AgELISA, red) and competitive ELISA (cELISA, yellow), respectively, in samples collected from captive Artiodactyla from Lunaret zoo (Montpellier, France; Table S1). AgELISA was performed on fecal samples using adapted method described in the text, and cELISA was carried on serum samples from the same animals. AgELISA results are expressed in Sample/Positive Control % (limit of detection < 20%), and cELISA in competition % (limit of detection > 50%) following the manufacturers’ instructions.

**
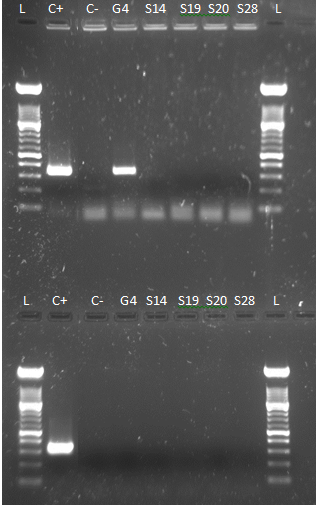
**

Qiagen

Quantabio

100bp

200bp

300bp

400bp

400bp

300bp

200bp

100bp

**Figure S4.** Comparative results for a RT-PCR amplifying a 351 base pair (bp) segment of the PPRV N gene using two different kits, Qiagen one step RT-PCR, and qScript_XLT_One-Step_RT-PCR Kit. Samples tested were fecal samples collected from goat (G) and sheep(S) in Tanzania, 2015.

**References**

1. Couacy-Hymann, E.*, et al.* Rapid and sensitive detection of peste des petits ruminants virus by a polymerase chain reaction assay. *J Virol Methods*. **100**, 17-25 (2002).

2. Kwiatek, O.*, et al.* Quantitative one-step real-time RT-PCR for the fast detection of the four genotypes of PPRV. *J Virol Methods*. **165**, 168-77 (2010).

3. Enchery, F.*, et al.* Development of a PPRV challenge model in goats and its use to assess the efficacy of a PPR Vaccine. *Vaccine* (accepted).
